# Satire without borders: the age-moderated effect of one-sided versus two-sided satire on hedonic experiences and patriotism

**DOI:** 10.1515/humor-2022-0047

**Published:** 2023-02-23

**Authors:** Mark Boukes, Heather L. LaMarre

**Affiliations:** Universiteit van Amsterdam, Amsterdam, Netherlands; Temple University, Philadelphia, USA

**Keywords:** comparative analysis, emotions, experiment, hedonic enjoyment, one-sided versus two-sided humor, patriotism, satire

## Abstract

The differential satire effects across domestic and foreign audiences are largely unknown; yet, this is of growing relevance as political satire increasingly reaches international audiences. A two-country experiment was conducted in which satirical stimuli from the Netherlands with either a one-sided (only targeting the United States) or two-sided humorous message (targeting both the U.S. and the Netherlands) was presented to a domestic (in-group) or foreign (out-group) audience. Specifically, this study examines political satire’s differential emotional and attitudinal impact on audiences located in the country-of-production (Netherlands) or abroad (U.S.). Results show that satire sidedness *uniformly* influenced hedonic enjoyment: compared to two-sided satire, one-sided satire elicited negative emotions and decreased positive emotions for both the in-group (Dutch) and the out-group (U.S.) audience. Yet, satire *differentially* affected patriotic attitudes. This effect was moderated by country and age: younger U.S. citizens became less patriotic after exposure to the one-sided satire that targeted their country and decreased their positive emotions; older U.S. citizens, in contrast, became more patriotic after exposure to this one-sided satire that particularly increased their negative emotions. The Dutch audience’s level of patriotism remained stable irrespective of satire sidedness. Altogether, this study demonstrates how humor type, country-of-reception, and age matter for satire effects.

## 1 Introduction

Research on humor, and political satire specifically, has been bounded to domestic audiences; most frequently focusing on U.S. satire about U.S. politics. While more research regarding satire has emerged in other continents (e.g., Boukes 2019; Mendiburo-Seguel et al. 2017; Msimanga et al. 2021; Shao and Liu 2019), these studies still focused solely on domestic audience effects (i.e., effects in the country where the satire was produced). While U.S. media products always found their way to foreign audiences (Thussu 2007), social media and streaming services have increased the likelihood for non-U.S. satire to also travel in the opposite direction.

This prompts questions regarding what effects take place when satire crosses national borders and whether domestic versus foreign audiences are influenced differently. The current study addressed these questions, using an experiment to compare effects of Dutch satire on viewers from the U.S. (foreign audience) and the Netherlands (domestic audience). Furthermore, we explicitly differentiated between a one-sided satire that only targets the other country (i.e., Dutch satire targeting the USA; making U.S. citizens the out-group) versus a two-sided mode of humor that simultaneously included self-mockery towards the in-group (the Dutch in this case) and jokes targeting the foreign out-group (U.S.).

Besides the in-group versus out-group comparison, we disentangle how effects vary across younger versus older audiences. Age is an important factor to consider as satire is particularly popular among youth. Although many satire studies were conducted with student samples (Burgers and Brugman 2022), specific theory about what makes youth a special audience often remained undiscussed. Moreover, conclusions have been mixed whether satire is particularly influential among the youth. Some argue that found effects might just be a side-effect of these young audiences being more *highly educated* among student samples (Burgers and Brugman 2022) rather than their (younger) age. Skurka et al. (2022) suggested that such age-moderations cast doubt on the generalizability of the overall satire literature, because so many satire studies were conducted among student samples. To understand the moderating potential of age for satire effects, we further theorized and empirically tested differences in satire appreciation and persuasiveness among younger versus older citizens.

### 1.1 Satire and hedonic enjoyment

Remarkably little scholarly attention has been paid to how satire consumption affects *media enjoyment* (Becker 2020; Weinmann and Vorderer 2018); satire has mainly been regarded as a political genre rather than as entertainment genre. Yet, many watch satire for entertainment-oriented motivations (Feldman 2013), and satirists often state that their primary intention is to make people laugh (not to inform or persuade). Research indeed demonstrated that satire influences entertainment experiences, such as mirth (Peifer 2018; Peifer and Landreville 2020) or hedonic response (Stewart 2011). However, satire might also cause feelings of offense (Daskal 2015).

This study explores *the conditions* under which people hedonically enjoy one-sided versus two-sided modes of political humor (see Becker and Anderson 2019). Emotions are at the roots of hedonic experiences (Higgins 2006): when people feel happiness, joy or laughter, hedonic experience is high (Oliver and Raney 2011). “Hedonic” is the Greek word for “sweet”, which refers to its association with pleasure and the avoidance of pain (Higgins 2006).

Thus, hedonic enjoyment can *also* be achieved by distracting people from their negative thoughts and feelings (Knobloch-Westerwick 2006; Zillmann 1988). As such, *reduction* of anger, worry or fear should also be considered hedonic enjoyment. Thus, hedonic enjoyment should be analyzed by distinguishing between positive affect and negative affect (Bartsch 2012); although the latter has been mostly overlooked in existing humor research (Ferguson and Ford 2008). Still, both can be independently elicited by humorous stimuli (McGraw and Warren 2010) and, thus, should be investigated as separate dimensions of hedonic enjoyment.

#### 1.1.1 One-sided versus two-sided satire and the impact on enjoyment

Various theoretical frameworks explain why people enjoy humor (Ferguson and Ford 2008). The Benign-Violation Hypothesis posits that humor requires a violation (e.g., of social/linguistic norms or personal dignity). However, this violation should be perceived as being *benign* to be enjoyed (McGraw and Warren 2010). Whereas violations normally elicit negative affect, they may cause laughter when the violation occurs in a setting that is perceived as safe, playful or non-serious (i.e., benign). How much hedonic enjoyment is elicited by satire, therefore, becomes conditional upon the mode of humor—some being more benign than others.

The current study focuses on differences in elicited hedonic enjoyment between one-sided versus two-sided forms of humor (see Becker and Anderson 2019). One-sided humor puts forward a message that is mainly targeting an out-group and is other-deprecatory; this type of humor is the most common on television (Van der Wal et al. 2020), and in political comedy specifically (Niven et al. 2003). In contrast, two-sided satire simultaneously includes other-directed and self-directed humor; thus targeting both the in-group and the out-group in one message.

Exposure to self-directed versus other-directed humor has been shown to activate different brain regions (Chan et al. 2018), potentially leading to different levels of hedonic enjoyment. Self-directed humor that targets the in-group (as presented in two-sided satire) establishes an equal relationship between message sender and receiver (Meyer 2000): self-deprecating humor is a basis for friendship, perceived as sympathetic (Greengross and Miller 2008; Lampert and Ervin-Tripp 2006), and appears as good natured compared to other-directed ridicule (Becker and Haller 2014). As such, it allows for shared laughter at one’s own follies (Lee et al. 2015). Two-sided humor in which multiple targets (including the in-group) are mocked is generally more surprising (i.e., causing more humorous incongruity) and, thus, provokes more laughter (Baumgartner et al. 2018).

Other-directed ridicule (e.g., targeting an out-group) is often more Juvenalian (Becker and Haller 2014), more fear-inducing, and more likely to cause conformity behavior (Janes and Olson 2000). Such negative effects not only occur among people targeted by the one-sided humor (out-group), but also “spills over” to the bystanders witnessing it (e.g., the in-group). Such one-sided humor that solely disparages an out-group (i.e., “the other”) signals a joint hostility and works differentiating (Stewart 2011). Rather than increasing source-liking, other-directed humor harms attitudes towards the satirized target (Baumgartner et al. 2018; Becker 2012; Becker and Haller 2014) and is perceived as less benign. Combining these insights about the fear-inducing nature of other-directed ridicule in one-sided satire (Janes and Olson 2000) and the appreciation of self-deprecating humor that simultaneously mocks the in-group (Greengross and Miller 2008) in two-sided satire, we expected the following:

**H_1_:**

*Exposure to one-sided satire* (a) *reduces positive emotion and* (b) *increases negative emotion compared to two-sided satire.*

#### 1.1.2 Satire sidedness, social identity, and enjoyment

While one-sided satire is expected to elicit less hedonic enjoyment than two-sided satire, the strength of this sidedness effect is likely conditional upon whether the audience identifies more with the satirized target of the one-sided message (i.e., as an out-group) or with the source of the message (i.e., being in-group). After all, superiority theory explains that humor may cause positive feelings through a downward comparison (Gruner 1997). By making fun of *others’* misfortune or disparaging an *unaffiliated* target, one might experience amusement via enhanced self-esteem (La Fave et al. 1973). This element of group comparison is also present in disposition theory (Zillmann and Cantor 1972), which predicts that people enjoy humor more when they hold a negative attitude towards the disparaged target. In line with social identity theory (Tajfel and Turner 1986), one could then predict that people particularly enjoy humorous disparagement when their in-group is *not* targeted. In contrast, people will experience less hedonic enjoyment when their social identity as out-group is threatened by the humorous message.

Linking this back to the Benign-Violation Hypothesis, people are more likely to perceive a violation as benign when they identify less with the satire target (McGraw and Warren 2010). Although message recipients will still recognize the violation, people feel relatively less threatened when it only satirizes a perceived out-group. Alternatively, a negative effect on hedonic enjoyment will likely occur when people’s personal identity is targeted by the one-side satire (without self-mockery). Specifically, being a member of the targeted group elicits a negative emotional response among this out-group (Ford et al. 2020; Vande Velde et al. 2018). By extension, one-sided satire might even cause feelings of anger when its message is counter-attitudinal (Chen et al. 2017).

A negative emotional response is *less* likely for two-sided satire in which multiple groups are simultaneously targeted, including the in-group of the satire producer. Two-sided joking is by itself perceived as less aggressive, more sympathetic (Lampert and Ervin-Tripp 2006), friendly (Greengross and Miller 2008), and more easily discounted as being “just a joke” (Becker and Anderson 2019). Two-sided satire, consequently, induces less negative emotion (Janes and Olson 2000) among both the out-group and the in-group.

Thus, people are less likely to perceive satire as funny when it targets their political identity (Becker 2014; Boukes et al. 2015; Peifer and Landreville 2020) or gender identity (Abrams and Bippus 2011). As opposed to using individual identity level, the current experiment categorizes people more collectively according to their country of residence. One-sided satire from another country (Netherlands) that targets one’s own nation (U.S.) should especially threaten this nationality aspect of their identity and, thus, restrain viewers’ hedonic entertainment experiences when compared to viewers from the country where the satire was produced (Janes and Olson 2000; McGraw and Warren 2010). Two-sided satire, by contrast, is likely to be perceived as good-natured and unthreatening (Greengross and Miller 2008; Lampert and Ervin-Tripp 2006), eliciting hedonic enjoyment among both the out-group and the in-group of the satirist (Stewart 2011). Accordingly, we expected a moderation effect wherein the negative effects of humor’s one-sidedness on hedonic enjoyment are stronger among the out-group that is directly targeted by the satire:

**H_2_:**

*Exposure to one-sided satire* (a) *reduces positive emotion and* (b) *evokes negative emotion compared to two-sided satire, and this effect is stronger among the out-group whose identity is targeted by the one-sided satire than among the in-group.*

#### 1.1.3 Satire sidedness, age, and enjoyment

Previous research found that younger audiences process satirical texts more easily than older audiences (Skalicky and Crossley 2019), and that younger audiences are more narratively absorbed when exposed to satire as opposed to news (Boukes et al. 2015). The differential way of processing satire by younger versus older viewers might also influence how their hedonic enjoyment experiences are affected by one-versus two-sided satire. When people are more narratively engaged with the storyline of media content, the impact on their emotional state will be arguably heavier (Murphy et al. 2013).

Because younger people are more engaged with satirical content than older people (Boukes et al. 2015; Skalicky and Crossley 2019), they will be more likely to experience a hedonic state that is congruent with the satire mode. A humorous appeal in an alarming climate change message, for example, elicited a higher perceived risk among younger compared to older viewers (Skurka et al. 2022), ostensibly due to stronger emotional responses. Accordingly, we expected a moderation by age with an even stronger negative effect of one-sided satire on hedonic enjoyment compared to two-sided satire among the younger viewers:

**H_3_:**

*Exposure to one-sided satire* (a) *reduces positive emotion and* (b) *evokes negative emotion compared to two-sided satire, and this effect is stronger among younger than among older people.*

### 1.2 Satire and patriotic attitudes

#### 1.2.1 Satire sidedness, social identity, and the impact on patriotism

The other-deprecating message in one-sided satire likely causes a stronger identity threat among the targeted out-group people compared to the in-group people who are not targeted by this one-sided satire (Lee et al. 2015). This difference between in-group and out-group is less likely to occur after exposure to a two-sided humorous message (Vande Velde et al. 2018).

Concretely, satire targeting one’s own country could pose a perceived threat to one’s national identity (Ford et al. 2020; McGraw and Warren 2010), which might be mitigated by attributing less importance to this identify (e.g., lowering patriotic attitudes). This is less likely to occur when people are confronted with a two-sided satirical message that is perceived as relatively benign and easily discounted (Becker and Anderson 2019). In such case, the one-sided satire message might weaken rather than strengthen self-identity among the out-group compared to two-sided satire.

However, the opposite is also possible. Identity threat caused by one-sided satire could instigate a process of motivated reasoning and counterarguing among the out-group, which eventually could reinforce their identity. To examine this, the current study operationalizes this potential process by studying *patriotism* as dependent variable.

From the literature, it is also not obvious whether one-sided satire or two-sided satire results in the highest level of patriotism among the in-group audience. When a political actor engages in self-directed humor, citizens generally develop a more positive attitude towards the politician (Baumgartner et al. 2018; Becker and Haller 2014). By engaging in two-sided humor and mocking oneself, people put themselves in a vulnerable position and demonstrate enough confidence to make jokes about one’s own shortcomings (Weisfeld 1993). Moreover, self-deprecating humor is typically associated with attractive personality traits (Greengross and Miller 2008; Lampert and Ervin-Tripp 2006). By extension, such two-sided humor likely increases audiences’ positive responses to the satire (i.e., by being proud of the production) potentially even strengthening the identity (i.e., patriotism) of those within the satirist’s in-group. Put simply, it is possible that two-sided humor disarms and creates positive affect among in-group audiences which, in turn, bolsters their in-group identity. If so, then the in-group’s patriotism could be positively influenced by the two-sided satire.

However, it is also true that this in-group is only confronted with criticism of their own identity within the two-sided satire. When combined with the persuasive power of satire (Burgers and Brugman 2022), this could work to weaken one’s self-perception. Thus, manifesting as relatively weaker patriotism. With regard to one-sided satire, moreover, the in-group can more easily engage in a downward comparison (see superiority theory, Gruner 1997) and feel better about themselves. Accordingly, the theoretical expectations are mixed and no prediction can be made about the general effect of satire sidedness on patriotism, especially whether this works the same or differently for the out-group and the in-group. Hence, we explored the following research question.

**RQ_1_:**

(a) *How does exposure to one-sided satire influence patriotic attitudes compared to exposure to two-sided satire, and* (b) *how does this effect differ between the in-group and the out-group?*

#### 1.2.2 Satire sidedness, age, and patriotism

The well-established Impressionable Years Hypothesis (Krosnick and Alwin 1989) states that political attitudes develop most strongly from late adolescence until early adulthood after which these attitudes tend to crystalize (Sears 1983). Young citizens are still developing their worldviews (Arnett et al. 2001), which also helps explain their attraction to satire that often presents clearcut opinions to (dis)agree with (Marchi 2012). Because younger audiences are still forming their political attitudes, it is quite probable that their social identities will be affected differently than older audiences. As such, the current study tests whether satire’s effect on patriotism is different for younger viewers when compared to older viewers.

Previous research found that younger people are more narratively engaged in satirical content than older viewers (Boukes et al. 2015). Narrative engagement, subsequently, results in stronger persuasive effects (Moyer-Gusé 2008; Slater and Rouner 2002) ostensibly because critically scrutinizing the message through motivated reasoning would restrain the enjoyment that audiences hope to achieve by consuming entertainment (Moyer-Gusé 2008). Hence, satire could more strongly influence the absorbed, younger audience (Landreville et al. 2010).

Accordingly, it is rather unlikely that younger foreign viewers (in our case from the U.S.) engage in counter-arguing to defend their social identity when exposed to one-sided satire from another country that targets their nation hence, their level of patriotism likely decreases. In contrast, older audiences will be relatively less engaged in the satirical narrative and therefore will likely experience more reactance, feeling more disparaged by the one-sided satire. In such case, we would expect the older audiences to counterargue the message, thereby potentially strengthening their patriotism to protect their self-identity.

Put simply, we expect that narrative engagement among younger audiences reduces their propensity to engage in identity protection in response to the harsher critique in one-sided satire, while more reactance (e.g., counter-arguing) among the relatively less engaged older audiences stimulates their need for identity protection. Thus, a cleaved age moderation effect would ensue. Previous research has also found that older citizens learn less from political comedy exposure than younger people (Cao 2008; Hollander 2005) as they probably find it a less legitimate source of information. Such discounting would also make it unlikely that older viewers let satire negatively influence their self-image. Accordingly, we expected a moderation effect with the impact of satire sidedness on the out-group of U.S. viewers being conditional upon age:

**H_4a_:**

*Exposure to one-sided satire that targets the out-group alone compared to two-sided satire that simultaneously targets both the in-group and the out-group will cause a stronger decrease of patriotism among younger out-group citizens than among older out-group citizens.*

Similarly, age was expected to moderate the effect of satire sidedness on patriotism among in-group citizens (in our case: Dutch viewers). Also for the in-group, a less critical processing of the satire would be expected among the younger audiences that will be more narratively engaged and therefore more easily persuaded (Boukes et al. 2015). Younger viewers will arguably be more responsive to and learn from the self-critique that is presented in the two-sided satire (Cao 2008; Hollander 2005), whereas critique on this in-group is not presented in the one-sided, other-directed satire. Older viewers of the in-group, in contrast, will be more likely to be offended by and counterargue the criticism on their self-identity that is presented in the two-sided satire. For the in-group of Dutch viewers, we accordingly expected:

**H_4b_:**

*Exposure to two-sided satire that simultaneously targets the in-group and the out-group compared to one-sided satire that targets the out-group alone will cause a stronger decrease of patriotism among younger in-group citizens than among older in-group citizens.*

## 2 Method

A randomized experiment was simultaneously conducted in the Netherlands and the United States using samples from *SSI/ResearchNow*. Quotas were set on age (*M* = 46.1 in U.S, *M* = 46.9 in Netherlands), gender (53% female in both countries), and political ideology to ensure a diverse sample on at least these characteristics. To ensure data quality, participants who did not recall the topic of the video (*n* = 37) or who were outliers on response time (following Tukey’s 1977 method based on relative cutoffs calculated with means of the interquartile range, *n* = 51) were excluded and dropped from the dataset *prior* to data analysis.1Initially, the cut-off point of 60 minutes or more was used as rule-of-thumb, leading to substantially the same results. However, Tukey’s method is a stronger standard in the literature and provides the necessary robustness against outliers at the tails (i.e., responses that took unusually long, see Berger and Kiefer 2021). This resulted in a final sample size of 492 participants (*n*_Netherlands_ = 247; *n*_USA_ = 245). Average response time was 21 min (SD = 6.89).

### 2.1 Stimuli

Participants were randomly exposed to one of two satire clips that lasted between 3 and 4 min: randomization was successful; hence, no control variables were needed in the statistical analyses.2No differences between conditions were found for age, *F*(1, 470) = 0.01, *p* = 0.942, gender χ^2^(1) = 1.26, *p* = 0.262, education, *F*(1, 470) = 1.88, *p* = 0.171, nor political ideology, *F*(1, 470) = 0.32, *p* = 0.570. Both videos were produced and broadcasted by the Dutch satire show *Zondag Met Lubach.* Participants were explicitly informed about the Dutch origin of the clip on the survey-page *before* stimulus exposure. The original videos were already narrated in English, which made them understandable for a foreign English-speaking audience (in this case: U.S. audience) and explains why the videos could go viral outside of the domestic Dutch context. Both videos satirized a conservative/right-wing political subject directly related to the United States.

#### 2.1.1 One-sided satire (*n* = 239)

The one-sided condition satirized the United States through the National Rifle Association of America (NRA)—a gun rights advocacy group.3See: https://www.youtube.com/watch?v=a-o9pwWUzz0. It criticized the weapon policy of the United States and describes a “*terrible epidemic: nonsensical rifle addiction, NRA.*” The video continues by claiming that NRA is a “*constitutional disorder that is caused by a dysfunction of the prefrontal second amendment in the nonsensical cortex causing patients to shoot. It starts with an innocent Colt, but soon patients will show signs of shotguns, sniper rifles, and M-16s even. Often, patients use silencers to hide their condition.*” After describing the danger for the environment of people with NRA, it stated that “*NRA is highly contagious. Parents often pass it on to their children: this happens automatically or semi-automatically.*” By the end of the clip, it showed a list of bullet points—because these bullets do not kill people – stating that the Red Cross can help the situation with “*water, blankets, facts, insights, statistics, and truth bombs.*” Importantly, the one-sided satire clip did not contain any reference to the Netherlands; it was fully other-directed.

#### 2.1.2 Two-sided satire (*n* = 233)

The second video introduced the Netherlands to Donald Trump upon his inauguration speech in which he emphasized “America First”.4See: https://www.youtube.com/watch?v=ELD2AwFN9Nc. It did so with two-sided jokes that simultaneously satirized the U.S. and its new President (and thereby the U.S. electorate) but also mocked the home country of the satirist (the Netherlands). The video ironically presented topics for which the Dutch could be similarly ‘proud’ as U.S. citizens. Examples included a major dam (“*great, great wall that we built to protect us from all the water from Mexico*”) and amusement park particularly known for its pony rides: “*The best pony park in the world. It’s true. They are the best ponies. You can ride them, you can date them, you can grab ‘em by the pony. It’s fantastic.*” The satire continues: “*In December, we have this scandalous tradition of Black Pete. It’s the most offensive, the most racist thing you have ever seen. You’ll love it, it’s great.*” And later on: “*We also have a disabled politician for you to make fun of (….), she is from the Ministery of Silly Walks.*” By the end of the clip, the narrator asked Trump for support: “*We totally understand it is going to be America first. But can we just say, the Netherlands second?*” Thus, this two-sided satire targeted both the other (i.e., U.S.) and the self (i.e., the Netherlands).

#### 2.1.3 Manipulation check

A χ^2^-test confirmed that the two clips functioned in the intended way regarding satire sidedness, χ^2^(4) = 251.78, *p* < 0.001: the one-sided satire clip was perceived by 82.8% of participants to solely be “criticizing (making fun of or joking about)” one country (i.e., the USA) only. The dominant perception in the two-sided satire condition was instead that the clip criticized the two nations simultaneously (81.5%).

### 2.2 Measurements5All multi-item scales were confirmed to load on only one latent component in principal axis factoring with oblique rotation.

#### 2.2.1 Dependent variables

***Hedonic enjoyment.*** Enjoyment was measured with two separate scales to represent that people may experience both positive and negative emotions independent from each other (Bartsch 2012; McGraw and Warren 2010) while viewing a satire clip. After exposure to the video, participants were asked how strongly they felt a list of discrete emotions while watching the video (scale: 0–10, items inspired by Richins 1997).

From this list of emotions, a scale of *positive emotion* was created consisting of three emotions (*α* = 0.74, *M* = 4.17, SD = 2.76): surprised, happy, proud. A scale of *negative emotion* was composed of the following emotions (*α* = 0.89, *M* = 2.76, SD = 2.60): angry, concerned, frustrated, afraid, offended. Positive and negative emotion only correlated weakly (*r* = 0.095, *p* = 0.040), which demonstrated that these are two separate constructs requiring separate analysis.

***Patriotism***. Four items from the scale of Huddy and Khatib (2007) tapped on a 0–4 range measured patriotism (*α* = 0.82, *M* = 2.10, SD = 0.96): (a) I support my country’s political leaders even if I disagree with their actions; (b) people who do not wholeheartedly support America/Netherlands should live elsewhere; (c) I believe that U.S./Dutch policies are almost always good for our country; and (d) America/Netherlands is a better country than most other countries.

#### 2.2.2 Moderator

***Age category***. Age of participants ranged from 19 to 67. The *PROCESS 3.3*-tool (Hayes 2013) was used to analyze the interaction effects with age as a moderating factor. To ease interpretation of the interaction effects, results of supplementary ANOVA analyses were plotted. For these plots, participants were categorized as belonging to either the “younger” (34 years old or younger, 26.9%) or “older” people (35 and above, 73.1%). The cut-off point of 34 years followed the boundary conditions for satire effects found in earlier research (Boukes et al. 2015); and this cut-off point was closely replicated in the current study.

***In-group versus Out-group.*** Whether people belonged to the in-group or out-group relative to the satire was determined with the sample from which they originated. 235 people participated from the United States (49.8%), they represented the theoretical out-group in this study. 237 people were recruited from the Netherlands (50.2%), who represented the theoretical in-group coming from the same country as where the satire had been produced.

## 3 Results

### 3.1 Hedonic enjoyment

#### 3.1.1 Main effect of satire sidedness on enjoyment

The sidedness of the satire stimuli affected audience emotions in the hypothesized direction. One-sided satire (*M* = 3.52, SD = 2.55) had a strong suppressing effect on the positive emotions that were experienced while viewing the clip compared to exposure to the two-sided satire condition (*M* = 4.84, SD = 2.57), *t*(470) = −5.60, *p* < 0.001, Cohen’s *d* = 0.52. This provides evidence in line with Hypothesis 1_a_.

Negative emotion was also strongly affected in the expected direction, *t*(470) = 3.66, *p* < 0.001, Cohen’s *d* = 0.34. One-sided satire (*M* = 3.19, SD = 2.47) evoked more negative emotion than exposure to two-sided satire (*M* = 2.32, SD = 2.64). This provided evidence in line with Hypothesis 1_b_. As can be inferred from the Cohen’s *d*-values, the effect of satire sidedness on positive emotions was stronger than the effect on negative emotions.

#### 3.1.2 Conditionality upon in-group versus out-group (hedonic enjoyment)

**Figure 1:**
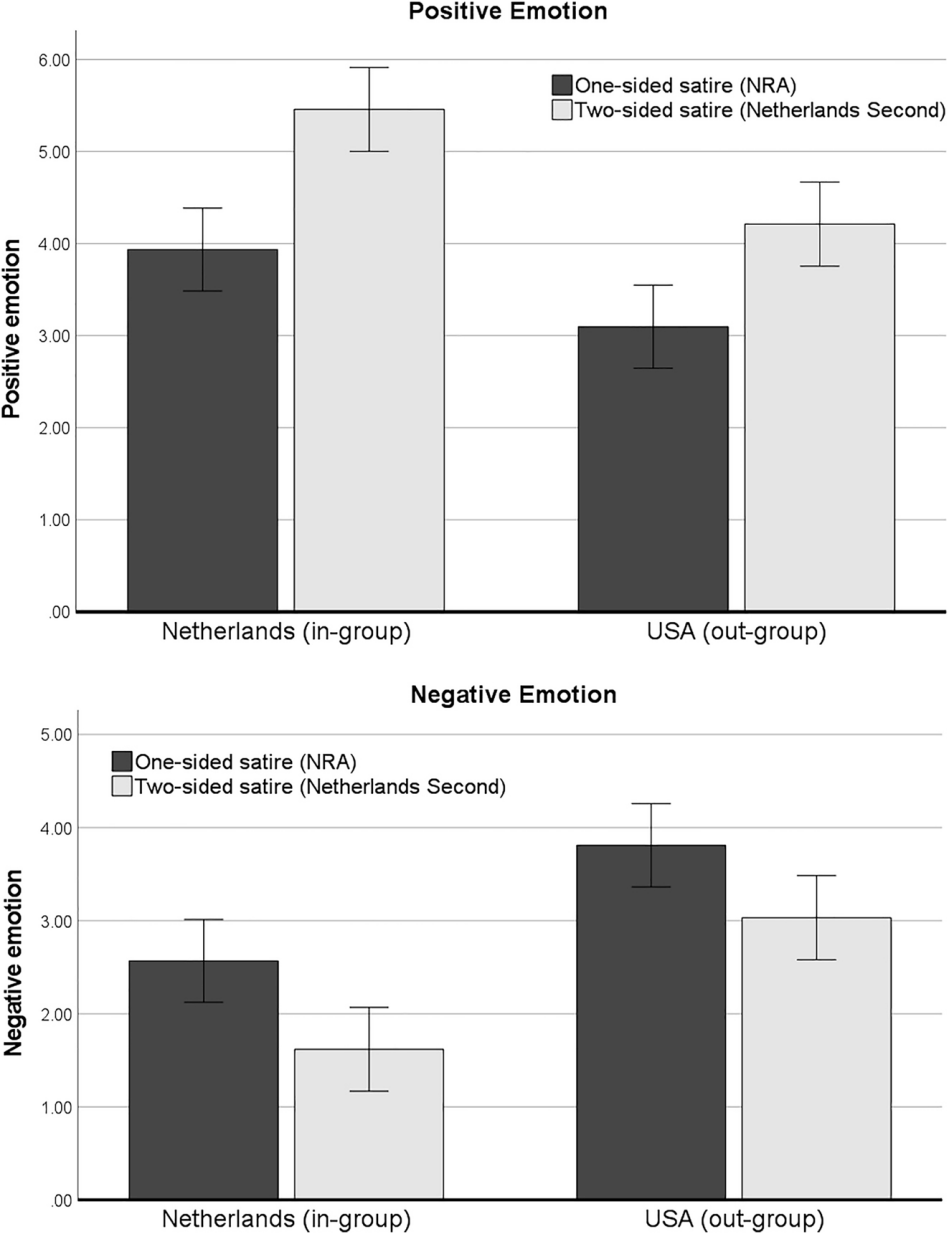
Estimated means and confidence intervals of positive emotion (above) and negative emotion (below) in the two satire conditions among the in-group (Dutch) and out-group (U.S.) sample.

The out-group viewers from the targeted country in the one-sided satire (United States) did not experience stronger decreases of positive emotion nor stronger increases of negative emotion compared to the in-group of Dutch viewers. The interaction effects between satire sidedness and the country from which participants originated were insignificant for both indicators of hedonic enjoyment: for positive emotion, *F*(1, 468) = 0.78, *p* = 0.377; and for negative emotion, *F*(1, 468) = 0.142, *p* = 0.706. Figure 1 visualizes the effects and shows that one-sided satire (the black bar) uniformly *reduced* positive emotion (graph above) for both the in-group of Dutch viewers and the out-group of U.S. participants compared to the two-sided satire. Moreover, the one-sided satire also uniformly *increased* negative emotion (graph below) among both the in-group and the out-group compared to the two-sided satire. Thus, no support was found for Hypothesis 2.

#### 3.1.3 Conditionality upon age (hedonic enjoyment)

We hypothesized that the emotions of younger viewers should be more strongly affected by the sidedness of satire compared to older viewers. Running a full factorial model (using Process v4.0’s Model 3, see Hayes 2013) with satire sidedness, age (continuous variable) and country as independent variables together with all their possible interaction effects, we yielded a significant three-way interaction effect on experienced positive emotion, *b* = −0.08, SE = 0.03, *p* = 0.017. This implies that the effect of satire sidedness on hedonic enjoyment was indeed conditional upon age, but that the strength of the moderation effect depended on the specific country.

**Figure 2:**
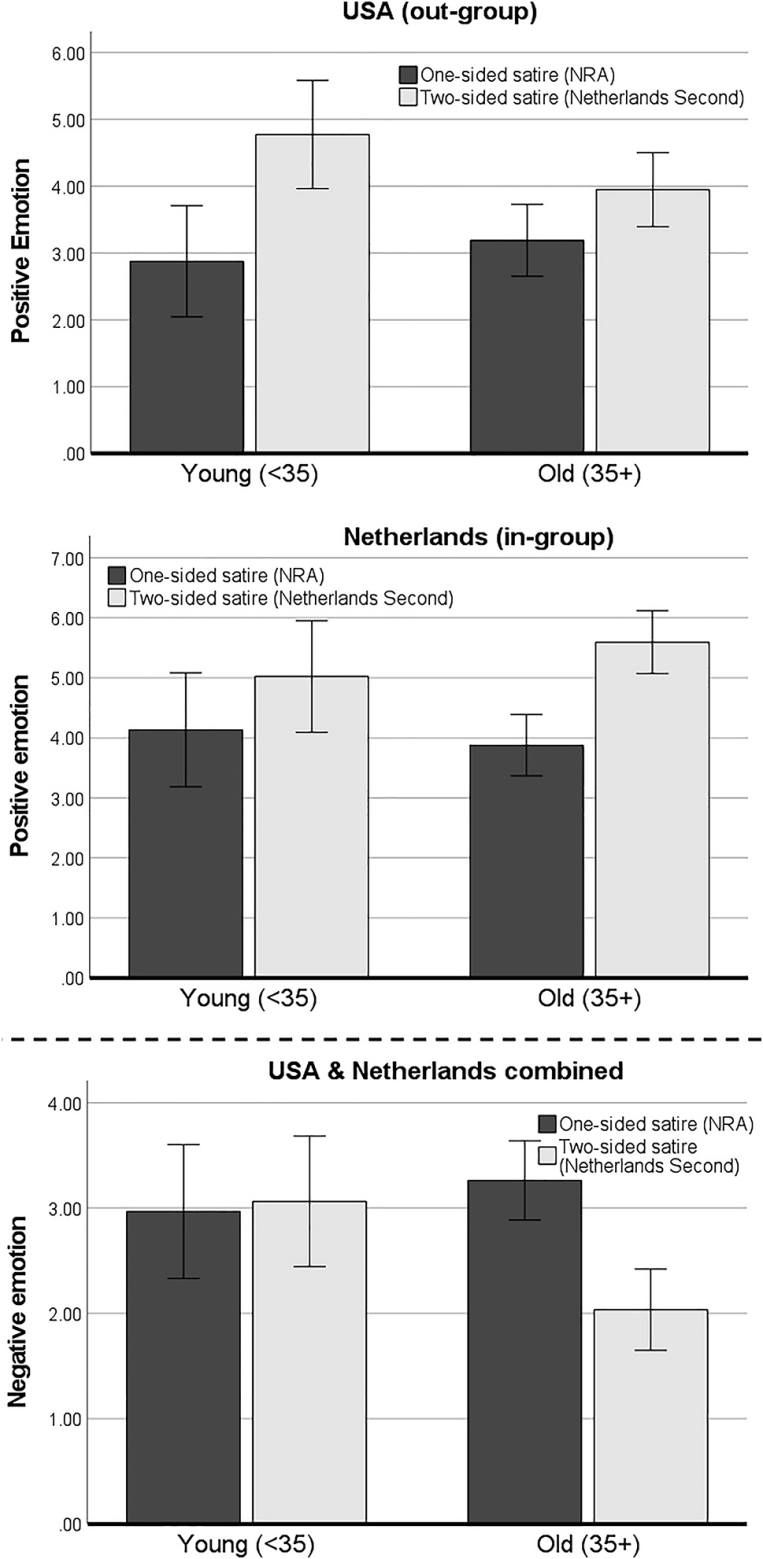
Estimated means and confidence intervals of positive and negative emotion for younger and older participants in the satire conditions among the U.S. and Dutch sub-sample.

Looking at the U.S sample (Figure 2, upper graph), we can conclude that the one-sided satire reduced positive affect most strongly among the younger viewers (e.g., 32 years old [−1 SD]: *b* = −1.59, *p* < 0.001) compared to the older viewers (e.g., 60 year-old [+1 SD]: *b* = −0.60, *p* = 0.159). The difference in effect strength was significant and in the expected direction: relatively less positive emotion was elicited by the one-sided satire versus the two-sided satire among the younger people.

An opposite pattern was found in the Netherlands (Figure 2, middle graph). In the Dutch sample, the negative effect of one-sided satire on positive emotion was slightly stronger among the older participants (e.g., 60 year-old [+1 SD]: *b* = −2.16, *p* < 0.001) than among the younger participants (e.g., 32 years old [−1 SD]: *b* = −0.87, *p* = 0.073). Hypothesis 3_a_ was thus only partially supported: one-sided satire indeed reduced positive emotion most strongly among younger viewers, but only in the satirized out-group of U.S. viewers.

With respect to negative emotion, the three-way interaction between satire sidedness, age, and country was insignificant (*p* = 0.741). Examining this interaction effect while leaving out the country factor, though, we found that the positive effect of one-sided satire on negative emotions strengthens with higher age, *b* = −0.05, SE = 0.02, *p* = 0.004. The Johnson-Neyman significance region begins at age 39: Figure 2 (lower graph) shows that no significant effect was found among the younger viewers (e.g., 32 years old [−1 SD]: *b* = −0.18, *p* = 0.580), whereas negative emotions significantly increased after exposure to the one-sided versus the two-sided satire for the older viewers (e.g., 60 years old [+1 SD]: *b* = 1.54, *p* < 0.001). So, Hypothesis 3_b_ was rejected: one-sided satire elicited negative emotions particularly among the *older* audience – and this was not moderated by country.

### 3.2 Patriotism

#### 3.2.1 Main effect of satire sidedness on patriotism

No across-the-board effect of satire sidedness on patriotism was yielded in response to RQ_1a_. The satire video to which people were exposed did not uniformly affect viewers’ level of patriotism, *t*(470) = −0.26, *p* = 0.799.

#### 3.2.2 Conditionality upon in-group versus out-group (patriotism)

No moderated effect between country and satire sidedness on patriotism was found either, *F*(1, 468) = 0.62, *p* = 0.686. The answer to RQ_1b_ was, thus, that exposure to one-sided satire versus two-sided satire did *not* influence patriotism *differently* between the in-group and out-group, because neither were influenced across-the-board.

#### 3.2.3 Conditionality upon age (patriotism)

A full factorial OLS regression model was run using satire sidedness (one-sided vs. two-sided satire), age, and country of residence together with all their possible interaction effects as independent variables and patriotism as dependent variable. A three-way interaction effect on patriotism was yielded, *b* = −0.02, SE = 0.01, *p* = 0.049. Zooming in on this finding, the interaction effect between satire sidedness and age on patriotism was only significant in the U.S. (*p* = 0.008), but not in the Netherlands (*p* = 0.816). Hypothesis 4_b_ is thus rejected, because that sub-hypothesis expected an interaction effect among the in-group of Dutch viewers. However, no effects of satire sidedness on patriotism were found among the in-group participants from the satire’s country of production (i.e., not for younger nor older citizens).

**Figure 3:**
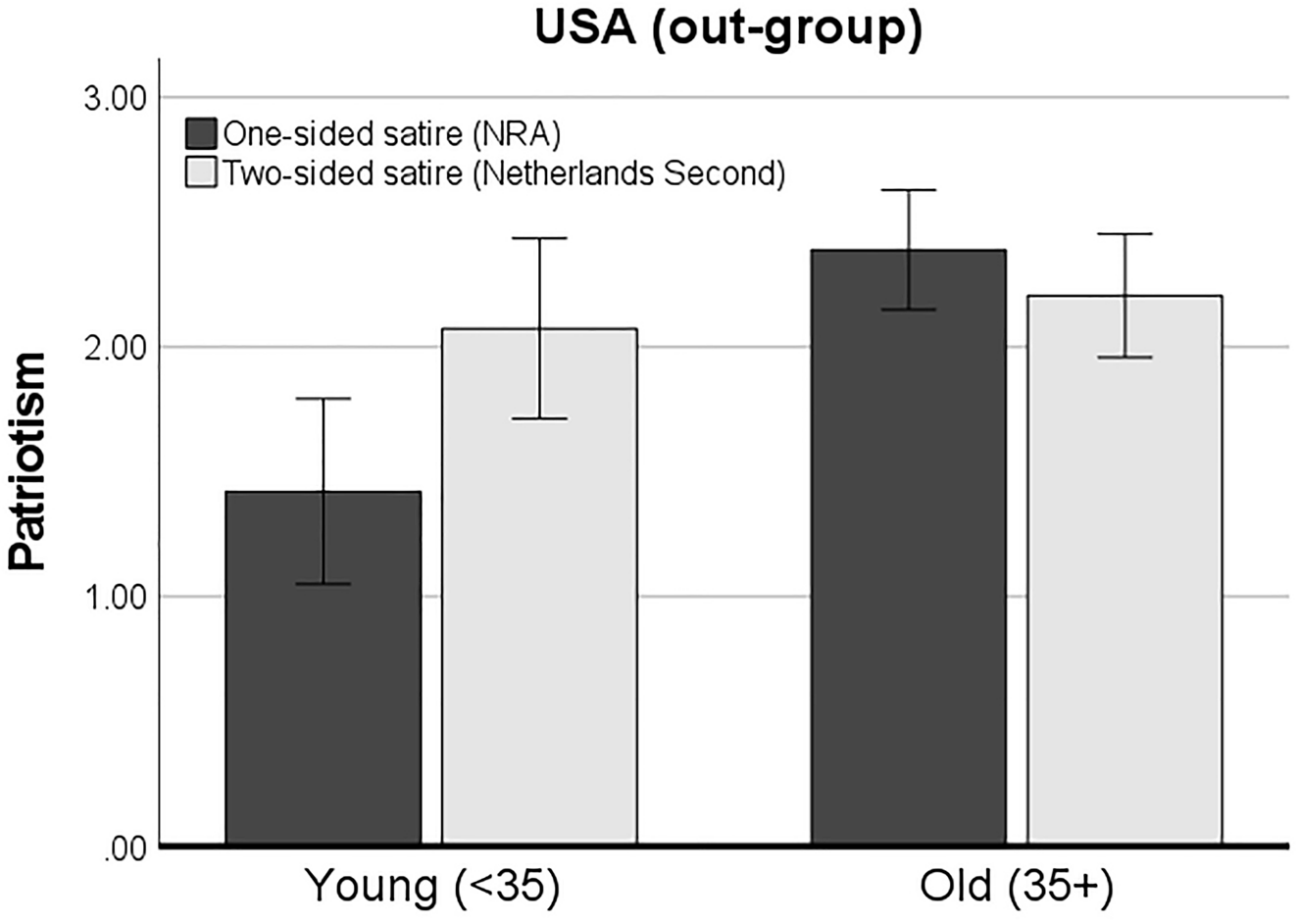
Estimated means and confidence intervals of patriotism in the satire conditions among the younger and older U.S. sub-sample.

Regarding the U.S. sample, an interesting pattern was found. Results showed that the effect of exposure to one-sided versus two-sided satire on patriotism indeed varied for younger versus older participants. The Johnson-Neyman significance region was found at value below 33.2 years old (for negative effect on patriotism) and above the value of 65.3 years (for a positive effect on patriotism); these age boundaries closely replicate the moderated effect in earlier work (Boukes et al. 2015). Figure 3 displays the results for the U.S. sample of an additional ANOVA analysis, which confirmed this interaction effect, *F*(1, 231) = 7.04, *p* = 0.009. As predicted, a small positive effect on patriotism was found in exposure to the one-sided satire for the *older* U.S. citizens; thus, indicating a potential process of reactance among the older out-group. This corresponds with the stronger negative emotion they experienced in response to the one-sided satire.

For younger U.S. citizens, in contrast, a negative effect of exposure to the one-sided satire on patriotism was found. Thus, indicating a process of message acceptance, which corresponds to their lower level of positive emotions after the one-sided satire. Altogether, this finding confirms Hypothesis 4_a_, which predicted that one-sided satire targeting the U.S. compared to two-sided satire would result in a higher level of patriotism among older U.S. citizens compared to younger U.S. citizens. The latter (young U.S. citizens) instead became less patriotic due to the one-sided humoristic attacks on their country.

## 4 Discussion

The present study examined the emotional and attitudinal impact of satire sidedness on domestic and foreign audiences. Thereby, we add a comparative perspective to the increasingly rich satire literature and expand existing insights on the different effects that one-sided satire and two-sided satire might have. Although it is unknown how many foreign produced satire is being consumed, such clips are regularly promoted by U.S. celebrities,6E.g., https://twitter.com/samuelljackson/status/966060490219597824. interacted with on social media (Boukes et al. 2022) and picked up by U.S. mass media (e.g., Donadio and Stack 2017; Lopez 2018). This altogether increases the reach to the non-domestic, U.S. audience (in this case, the out-group) and creates possibilities for impacting a foreign out-group. Recent initiatives, such as *Netflix* “Comedians of the World”-series (Husband 2018), will only further boost the global viewership of satire products. These developments increase the relevance of the current study’s findings and point to an increasing need for understanding satire effects on non-domestic audiences.

Specifically, we investigated the effects of Dutch produced satire about the United States on hedonic enjoyment and patriotism among Dutch and U.S. audiences, as well as how this differs among younger versus older people. Regarding hedonic enjoyment, we confirm the hypothesis that one-sided satire across-the-board elicits less positive and more negative emotions compared to two-sided satire. Alternatively, one could interpret that two-sided satire is most successful in generating hedonic enjoyment.

Surprisingly, the effects of satire sidedness on hedonic enjoyment were not conditional upon whether viewers belonged to the in-group or out-group (i.e., country of residence) alone. This suggests that it is not just the (downward) social comparison in one-sided satire that restricts the process toward enjoyment (e.g., Ford et al. 2020; Gruner 1997). Instead, the across-the-board effect could be caused by the fear-inducing nature of one-sided humor that is also experienced by the in-group who are not targeted by the satire (Janes and Olson 2000). Moreover, the more surprising (Baumgartner et al. 2018), sympathetic (Greengross and Miller 2008), and easily discounted nature of two-sided satire messages (Becker and Anderson 2019) potentially made it likely to be enjoyed by both groups.

With respect to patriotism, we have not found an omnibus effect of satire sidedness. A cleaved moderation effect (see Holbert and Park 2020) of satire sidedness by age has been found on patriotism, but only among the out-group of U.S. viewers. In particular, younger audiences from the U.S. expressed *lower* levels of patriotism after exposure to the one-sided satire compared to the two-sided satire. Older U.S. audiences, in contrast, maintained a relatively *higher* level of patriotism after exposure to the one-sided satire that criticized their country. Two theoretical explanations help to understand this conditional response in the non-domestic out-group. First, younger audiences probably were more narratively engaged with the story line of the satire (Boukes et al. 2015), thereby creating less resistance to the critical messages about their country (Moyer-Gusé 2008) and subsequently decreasing their patriotism. Second, the older audiences who arguably were less narratively engaged could, therefore, have experienced relatively more identity threat and, hence, engaged in self-bolstering thoughts to maintain their sense of national pride.

**Figure 4:**
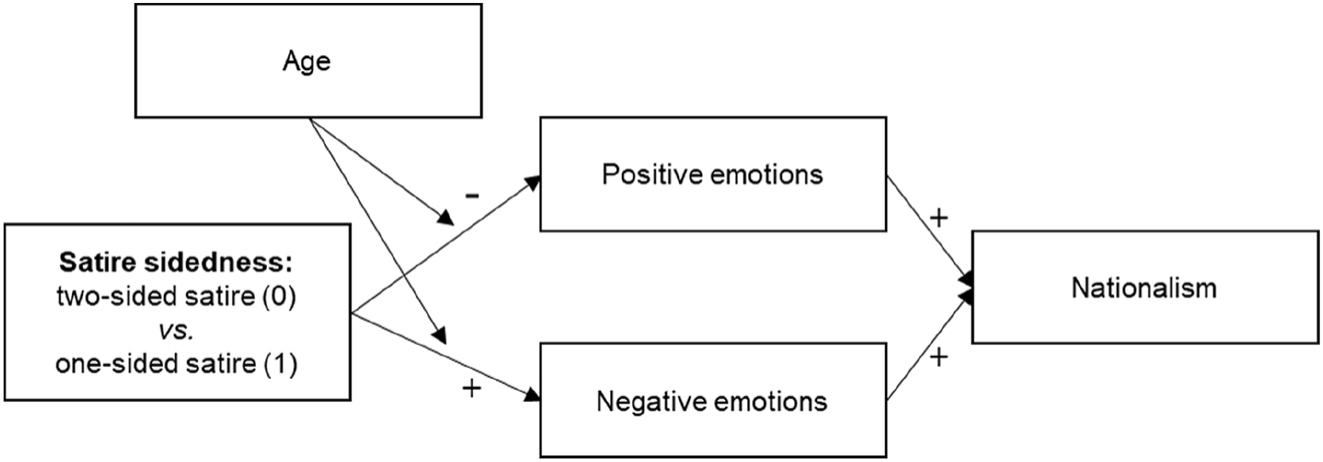
Moderated mediation model explaining nationalism through emotional response.

Additional analyses of emotional response as a mediating mechanism between satire sidedness and patriotism confirmed that both outcome variables could have influenced each other (see Figure 4).7For reasons of space not included in manuscript. For details, please see: https://osf.io/juzr8/?view_only=3434a33db4e1428fa8c4f52810ee4444. These analyses show that younger U.S. viewers experienced *drops* in positive emotion (happiness, pride) when exposed to the one-sided satire. In contrast, negative emotions (anger, fear, frustration) were particularly elicited among older U.S. citizens by the one-sided satire, which suggests that a motivated reasoning process was activated amongst them. Analyses of indirect effects, subsequently, showed that both the negative and positive emotions functioned as significant mediators to shape patriotic attitudes. Whereas patriotism *decreased* among young U.S. audiences due to message-acceptance of the one-sided satire that targeted their home country (via, less positive emotion), the opposite occurred for the older U.S. viewers who ostensibly engaged in motivated reasoning (via elicited negative emotion) to defend their national pride when confronted with the one-sided satire critiquing their nation. Exploratory analyses showed that the interactions with age have *not* been caused by differences in political ideology (no interactions with a conservative-liberal ideology scale).

The current study thus shows that context matters when predicting the effects of satire. Moreover, we have further explored the boundary conditions of the genre’s impact: when satire reaches a foreign audience with comedy that targets another nation, it is especially likely the younger foreign audience’s attitudes will be influenced in line with the satirical message, whereas the older foreign audience is more likely to experience a boomerang effect. Meanwhile, the domestic audience’s patriotic attitudes remained unaffected. This finding is especially relevant during a time when questions of foreign influence on domestic elections are rising throughout the world. Does exported satire have the power and influence to affect democratic processes in foreign nations? While likely constrained by reach and viewership, the potential for such influence merits further investigation.

Just as many studies on satire before, the current investigation tried to strike a balance between internal and external validity of the study design. This is always complicated for satire research, because externally valid stimuli (a) require high-quality audiovisual materials that (b) are still sufficiently humorous. This is difficult to craft oneself, making that most experimental studies (including our own) relied on existing clips. Consequently, one must compromise on internal validity: often studies compared clips of different shows with different hosts, which could be confounding factors. In our case, video clips have been used of the same show and of a similar length, but the targets of satire were different (NRA, Trump). Although these targets were consciously chosen for their relative ideological similarity, the target is still a potentially confounding factor, and thus a limitation, of our experiment. To elevate political satire research to a next level, future research should consider producing stimuli in cooperation with professional satire makers to create funny though comparable clips of different humor styles.
